# Management of Postpartum Haemorrhage

**DOI:** 10.5152/TJAR.2022.21438

**Published:** 2022-12-01

**Authors:** Berrin Günaydın

**Affiliations:** 1 Department of Anaesthesiology and Reanimation, Gazi University Faculty of Medicine, Ankara, Turkey

**Keywords:** Algorithm, guideline, management, obstetrics, postpartum haemorrhage

## Abstract

Postpartum haemorrhage is the leading cause of maternal mortality worldwide. However, postpartum haemorrhage-related deaths are potentially preventable with timely diagnosis and management. The present review addresses the management of postpartum haemorrhage algorithm that includes the use of uterotonics, non-surgical (balloon tamponade) or surgical (sutures, artery ligations, and/or hysterectomy) techniques, and/or endovascular radiologic interventions, antifibrinolytic (tranexamic acid), and procoagulant (fibrinogen concentrate) drugs based on the international and national guidelines updated with recent evidences.

**Main Points**

- Postpartum hemorrhage remains a clinically significant cause of maternal complications and death worldwide.
- To decrease maternal mortality, using a postpartum hemorrhage algorithm with trigger and target values is recommended.
- Management should include surgical/nonsurgical techniques/maneuvers along with evidence based use of massive transfusion protocol modified according to the facility needs/resources including hemostatic and/or pro-coagulant agents under the guidance of standard and/or point of care tests by multidisciplinary approach.

## Introduction

The aim of this present review is to address the management of postpartum haemorrhage (PPH) based on the international and national guidelines updated with recent evidence.

### Definition

According to the American College of Obstetricians and Gynaecolgists, PPH is defined as a cumulative blood loss greater than or equal to 1000 mL or blood loss accompanied by signs or symptoms of hypovolaemia within 24 hours after the birth process (includes intrapartum loss) regardless of route of delivery.^1^ The PPH is categorised as either primary or secondary; primary occurs in the first 24 hours after delivery (early PPH) and secondary PPH occurs 24 hours to 12 weeks after delivery (late or delayed PPH).^2^ According to global, regional, and national levels of maternal mortality rate (MMR) between 1990 and 2013, a systematic analysis of the global burden of disease study showed that 292 982 (95% UI: 261 017-327 792) maternal deaths occurred in 2013, compared with 376 034 (343 483-407 574) in 1990. Globally, the biggest absolute reduction was observed in death rates due to maternal haemorrhage from 71 295 (95% UI: 64 562-78 329) in 1990 to 44 190 (38 273-50 819) in 2013.^3^ The MMR has been most commonly resulted from PPH in Turkey with a rate of 13.1 in 100 000 live births in 2019.^4^ Therefore, vigilance and awareness about risk factors are very important before delivery.

### Risk Factors

According to the multidisciplinary consensus statement, the prediction of PPH is difficult and there is no single risk factor except abnormal placentation. Despite number of contributing risk factors (multiple pregnancies, history of PPH, pregnancy-induced hypertension, chorioamnionitis, episiotomy, pre-labour caesarean section, macrosomia, and operative vaginal delivery), PPH can develop unpredictably without any risk factor.^2^ Therefore, any maternal unit should be prepared to manage PPH with physicians in charge of maternal care. Main causes of PPH are represented by 4Ts (tone 70%, tissue 20%, trauma 10%, and thrombin <1%) which should be checked during management, and treatment should be directed accordingly. Uterine atony is the most common cause of PPH, and others include genital tract trauma, uterine rupture, retained placental tissue, and maternal coagulation disorders.^2^

### General Principles of Postpartum Haemorrhage Management

Management of PPH necessitates a coordinated multidisciplinary approach which involves good communication including an obstetrician–perinatologist, anaesthesiologist, haematologist–blood bank and interventional radiologist, for accurate assessment of blood loss, monitoring of maternal signs and symptoms, fluid, blood and blood product replacement, and arrest of the source of the haemorrhage.^5^

### Assessment of Blood Loss

Measuring blood loss as accurately as possible is essential for identifying the severity of haemorrhage and assessing patient’s response to haemorrhage. According to a pictorial reference guide to aid visual estimation of blood loss, the amount of blood estimations are soiled sanitary towel (30 mL), soaked sanitary towel (100 mL), small soaked swab 10 × 10 (60 mL), large soaked swab 45 × 45 (350 mL), and full kidney dish (500 mL). Additionally, blood in aspiratory tubes (includes amnion fluid as well) under buttocks drapes and surgery site (blood pooling) needs to be considered.^6^ However, in visual estimations, rate of misdiagnosis is around 35%-50%. Therefore, instead of estimated blood loss (EBL), since it is always very difficult as well as late, quantitative measurement is recommended. The following equation is used when calculating blood loss of a blood-soaked item: WET item gram weight – DRY item weight = millilitres of blood within the item.

For births with no rupture of the amniotic sac, the following volumes can be used to estimate the contribution of amniotic fluid in the aspiratory tubes; at term, it is approximately 700 mL, where it could be 300 mL or 1400 mL in oligohydramnios and polyhydramnios, respectively, as indicated.^7^

### Algorithm in Postpartum Haemorrhage Management

The PPH management protocol primarily includes the use of uterotonics (oxytocin, methylergonovine, carboprost or misoprostol), non-surgical (balloon tamponade) or surgical (compression sutures, internal iliac artery–ovarian–uterine artery ligations and/or peripartum hysterectomy) techniques, endovascular interventions (e.g., uterine artery embolization), antifibrinolytic (tranexamic acid [TXA]), and procoagulant (fibrinogen concentrate [FC]) administration as indicated both in European and our recently launched national guidelines entitled “Patient Blood Management and Maternity and Obstetrics.”^2,5,8-10^

### Bimanual Uterine Massage

It is usually the first step in the management of PPH due to uterine atony which is under the responsibility of obstetricians. Massage is performed in an attempt to induce uterine contractions by stimulating endogenous prostaglandins which are started simultaneously with uterotonics.^11^

### Uterotonics Algorithm

Administration of oxytocin is the mainstay of treatment for controlling PPH due to uterine atony and is usually commenced simultaneously with uterine massage.^11^ Optimal oxytocin dosage and route of administration during uterine atony are important to treat PPH and minimise side effects: intravenous (IV) 10-40 IU/500-1000 mL of lactated Ringer’s solution or intramuscular (IM) 5-10 IU for up to 4 doses. Undesirable effects of oxytocin include flushing, nausea and vomiting, tachycardia, hypotension, delayed water retention, hyponatraemia, and seizures. In case of inadequate control of postpartum haemorrhage with oxytocin, additional uterotonics such as methylergonovine, carboprost (15-methyl analogue of prostaglandin F2 alpha), or misoprostol (prostaglandin E_1_ analogue) have been shown to be effective as second-line uterotonics. However, there is a lack of evidence that suggests which specific additional uterotonics are the most effective.^12^

### Fluid Management

In bleeding situations, replacement of extracellular fluid losses with isotonic crystalloids in a timely protocol-based manner is suggested. Infusion of colloids provides haemodynamic stabilisation with less tissue oedema when compared with crystalloids. Dilutional coagulopathy can be aggravated with aggressive volume loading in severe bleeding. Therefore, restrictive volume replacement (1-2 mL crystalloid for each 1 mL blood loss) to avoid secondary hypervolemia and dilution of coagulation factors that would further aggravate coagulopathy are of utmost importance. However, maintaining normovolaemia is crucial to compensate for the losses and provide haemodynamic stabilization in this patient population.^5,8^

### Monitoring

Initial maternal monitoring includes heart rate (HR) and electrocardiography (ECG), blood pressure (BP), respiratory rate, and peripheral oxygen saturation followed by body temperature and urine output monitoring. Dynamic assessment of fluid responsiveness like stroke volume and pulse pressure variation, pleth variability index, and non-invasive cardiac output should be considered for the optimisation of fluid guidance during severe bleeding.^8^ Simply passive leg raising and fluid challenge tests have been introduced as one of many strategies to predict fluid responsiveness.^2,8^ Invasive BP (via an arterial line), central venous pressure (CVP), oxygen saturation, and cardiac output (via central line) are considered according to the severity of PPH and availability.^5^ However, monitoring of CVP and pulmonary artery occlusion pressure are no longer routine preferred techniques to guide fluid therapy because of their limitations.^8^

### Anaesthetic Management

Goals of anaesthetic management before peripartum hysterectomy include rapid assessment of the parturient to initiate appropriate monitoring, to restore intravascular volume, to achieve normovolaemia and haemodynamic stability, and to provide adequate anaesthesia and analgesia.

Maintaining normovolaemia and sustaining haemodynamics can be provided by infusing warmed isotonic crystalloids and transfusing blood/blood products using both non-invasive (ECG, HR, BP, peripheral oxygen saturation, body temperature, and cardiac output) and invasive monitorisation (arterial/central). Considering to maintain mean arterial pressure of 55-65 mmHg during the active bleeding phase of severe PPH until control of PPH is suggested.^5,8,9^

### Use of Blood/Blood Products

Although there are no precise criteria for starting blood transfusion in cases of PPH, transfusion is typically commenced when the EBL exceeds 1500 mL or when haemodynamic changes become apparent (Table 1).^1,13^

Estimation of blood loss by checking/measuring blood-soaked gauzes, sponges, sanitary pads, towels, aspiratory tubes, and drains and use of obstetric shock index derived by HR/systolic BP to start transfusion are helpful tools.^5^

**Figure 1.**
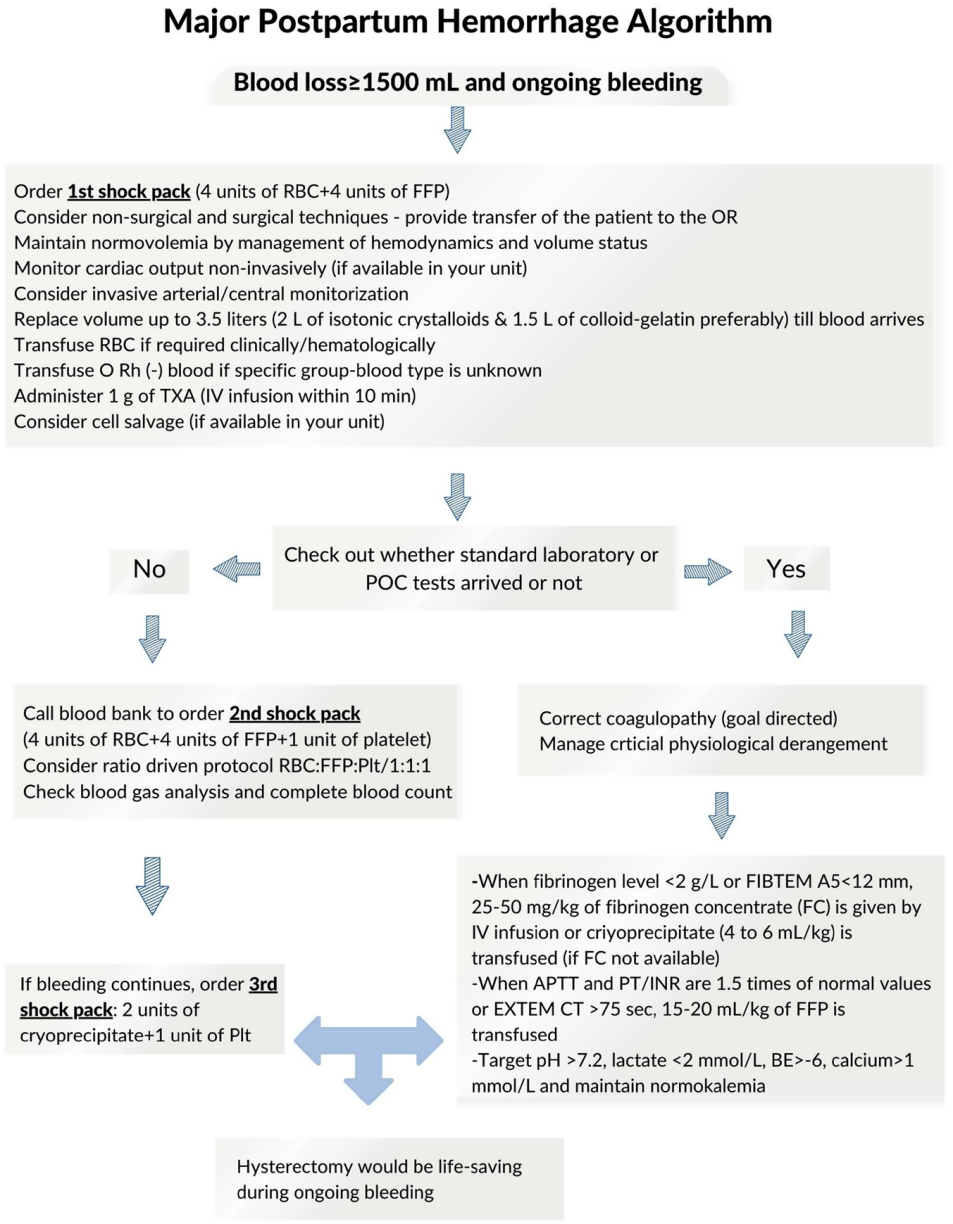
Major postpartum haemorrhage (PPH) algorithm.

Restrictive red blood cell (RBC) transfusion trigger of Hb <7 g dL^−1^ is preferred during the non-massively bleeding phase, but in case of active bleeding trigger of Hb level of 7-9 g dL^−1^, it can be changed depending on local protocols and coexisting maternal conditions. Transfusion of fresh frozen plasma (FFP) (15-20 mL kg^−1^) and platelet concentration (5-10 mL kg^−1^) should be based on the thresholds (Table 2). Physiologic acid–base status and calcium homeostasis are provided. Calcium drops during massive transfusion due to citrate-induced calcium binding should be monitored and corrected accordingly in severe PPH. This would optimise not only coagulation but also uterine muscle contraction. Preventive measures are taken against hypothermia since even mild hypothermia can increase blood loss (Figure 1).^9^

At the onset of major obstetric haemorrhage, if EBL is ≥1500 mL, first shock pack that consists of blood type-group specific 4 units of RBC + 4 units of FFP is ordered and then, step-down algorithm is followed. After checking laboratory test results, the use of blood/blood products (RBC, FFP, platelet concentration, and cryoprecipitate) is to be considered as second and third shock packs (Figure 1).^9^

In case of diagnosing PPH, one should call for help and activate blue code at first and start resuscitation measures of airway, breathing, circulation, defibrillation, and environment). If there is a high risk for the development of PPH (because of placenta previa, placenta accreta spectrum, or active vaginal bleeding), 2 large bore IV cannulas should be inserted. A specimen is collected for obtaining complete blood count and cross match. Blood bank is notified to be prepared for sending at least 2 units of blood-typed and cross-matched preferably (or 2 units of O Rh-negative blood if the blood type is unknown).^2,5^ If the patient is carrying high risk for PPH, central and arterial lines should be placed, but its routine use is not recommended.^2,8^

Secondly, the obstetrical protocols for transfusion of RBC, FFP, and platelets in a ratio of 6:4:1, 4:4:1, or 1:1:1 were derived from the trauma literature, as no clear data exist on the ratio for transfusing blood products in obstetrics. In case of massive bleeding, a ratio-driven protocol is advocated as soon as possible according to the European Society of Anaesthesiology Guidelines for the treatment of acquired coagulation factor deficiency.^8^ Coagulopathy treatment is guided by either standard laboratory tests or point-of-care (POC) tests (rotational thromboelastometry [ROTEM] or thromboelastography [TEG]) based on their readiness and/or availability.^9^

### Management of Hypofibrinogenaemia

Fibrinogen levels should be monitored as soon as possible either by standard laboratory tests or ROTEM/TEG in severe ongoing PPH. The decrease in fibrinogen is an early predictor of the severity of PPH and poor outcome. A plasma fibrinogen level <2 g L^−1^ threshold has been reported to have a predictive value of 100% for progression to severe bleeding.^14^

After introduction of ROTEM-guided algorithm in a large UK tertiary care referral unit, 110 out of 203 patients who had an EBL exceeding 1500 mL having FIBTEM A5 ≤12 mm received fibrinogen concentrate based on ROTEM-guided algorithm and those were compared with 52 patients and who met the same criteria received shock pack over a 12-month pre-intervention period. As a result of this study, the incidence of transfusion-associated circulatory overload, being one of the most important maternal complications, in the conventional shock pack group was found to be significantly higher than that of the algorithm group. The quantity of blood components (RBC, FFP, cryoprecipitate, platelet, and fibrinogen) was significantly higher in the retrospective shock pack group, compared to prospective algorithm group.^15^ In ongoing massive bleeding when EBL exceeds 1500 mL and fibrinogen level <2 g L^−1^ or FIBTEM A5 <12 mm, hypofibrinogenaemia should be corrected based on the PPH algorithm as presented (Table 2). Considering the treatment of hypofibrinogenaemia according to PPH algorithm, either FC of 25-50 mg kg^−1^ (1 g diluted in 50 mL injection water) is given by IV infusion or cryoprecipitate (4-6 mL kg^−1^) is transfused (Figure 1).^8,9,15^

The FIDEL trial aimed to test whether early administration of a 3 g FC given in patients experiencing PPH and resistant to oxytocin using a randomised double-blind multicentre (30 hospitals) design would be better management or not. Patients (n = 437) with minimum 4 g dL^−1^ reduction in haemoglobin and required minimum 2 units of RBC within 48 hours were enrolled in the study and then, randomly assigned to receive either 3 g of fibrinogen (n = 224) or placebo (n = 213) 30 minutes after switching to prostaglandin E2 analogue. Early systematic use of fibrinogen did not reduce blood loss, need for transfusion, and postpartum anaemia but prevented further hypofibrinogenaemia without increased risk of thromboembolism. Thus, current scientific evidence of the FIDEL trial is insufficient to support the use of FC in a routine blind setting.^16^ Another multicentre prospective cohort study investigated the clinical value of early viscoelastometric POC testing (ROTEM®FIBTEM) with amplitude of clot firmness at 5 min (A5) for predicting the progression of severe PPH and compare its predictive value with that of fibrinogen. Both fibrinogen concentrations and FIBTEM A5 values were determined between 800 and 1000 mL of blood loss within 24 hours of birth. Of recruited women (n = 391), severe PPH developed in 72 women with FIBTEM A5 of ≤12 mm and in 70 women with fibrinogen ≤ 2 g dL^−1^. Fibrinogen concentrations of ≤2, 2.1-3, 3.1-4, and >4 g dL^−1^ corresponding to FIBTEM A5 value of ≤12, 13-15, 16-22, and ≥23 mm were compared. So, in that study, positive predictive values for progression to severe PPH for FIBTEM A5 ≤12 mm and fibrinogen ≤2 g dL^−1^ were found to be 22.5% and 50%, respectively. Because of low predictive values, it was concluded that clinical value of ROTEM® FIBTEM A5 for predicting the severity of bleeding when routinely measured during the onset of PPH was reported to be limited. However, it was also reported that FIBTEM A5 might still be a promising POC test with clinical advantages in women with ongoing haemorrhage exceeding 1500 mL of blood loss.^17^

### Tranexamic Acid

The TXA is a licensed antifibrinolytic drug forthe treatment of peripartum and/or postoperative bleeding and DIC. One gram of IV TXA infused for the treatment of PPH has reduced deaths due to bleeding with a greater survival benefit when given within 3 hours of onset of PPH as reported in WOMAN trial.^10^ Therefore, early use of TXA (1 g diluted in 100 mL saline) by IV infusion within 10 minutes is highly recommended. It can be repeated 30 minutes after the first dose in ongoing bleeding.^9,10^ The TXA is also recommended to prevent bleeding during major surgery and/or to treat hyperfibrinolysis (20-25 mg kg^−1^).^8^

### Recombinant Activated Factor VII

Recombinant activated factor VII (rFVIIa) activates clotting cascade by cleaving factors IX and X which activates them and leads to the activation of thrombin and fibrin. However, there are some concerns with its use in severe PPH that leads to severe anaemia, thrombocytopenia, and hyperfibrinogenaemia resulting in thromboembolic events, cerebrovascular, and myocardial infarction.^18^ An initial dose of 60 µg kg^−1^ or 90 µg kg^−1^ is suggested based on the European and our national guidelines, respectively.^8,9^

In a study where local major PPH guidelines were prepared according to initial empirical off-label use of rFVIIa, 38 parturients who lost 1.5 times of her blood volume were treated with (n = 26) or without rFVIIa (n = 22). In patients receiving rFVIIa, total amount of blood loss was higher, activated partial tromboplastine time (APTT) and prothrombin time (PT) were longer, and fibrinogen level was significantly lower. Therefore, these patients required more RBC, platelet, and FC administration. These results did not give any evidence to extend the use of rFVIIa into less severe cases of PPH or into prophylactic use.^19^ Therefore, off-label administration of rFVIIa may be considered after correcting acidosis, hypothermia, and thrombocytopenia only in maternity patients with life-threatening haemorrhage when conventional measures including surgical hemostasis and appropriate blood component therapy failed (Table 2).^8,9^

### Cell Salvage

Data from literature demonstrated that cell salvaged blood is more physiological than allogenic blood since normal coagulation maintained without little or no additional clotting factors after its use. Even though the routine use of intraoperative cell salvage to reduce allogenic blood transfusion during caesarean sections has a cost, safe and economical use has been reported to be possible according to an observational data from almost a decade.^20-23^ In a multicentre (26 obstetric units) randomised controlled trial (SALVO) comparing routine use of cell salvage during caesarean section in women at risk of haemorrhage with current standard of care where cell salvage is not routinely used, rate of donor blood transfusion (primary outcome) was similar between the groups (3.5% in the control group n = 1492 vs. 2.5% in the intervention group n = 1498, *P*  = .056).^20^ Recently, McLoughlin et al^22^ suggested that routine cell salvage has been a more effective strategy than standard care in avoiding a donor blood transfusion in terms of relative cost and cost-effectiveness.

Network for the Advancement of Patient Blood Management, Haemostasis and Thrombosis (NATA) consensus statement recommends that intraoperative cell salvage should be available in high-level maternal care centres and be used in cases of massive PPH. Standard intraoperative cell salvage and reinfusion procedures should be used as part of the resuscitation strategy while remembering that intraoperative cell salvage output for reinfusion consists of predominantly erythrocytes. Reinfusion can be considered if the Hb drops below 8-10 g dL^−1^ or if blood loss increases from 800 to 1000 mL.^5^ As indicated in other guidelines, cell salvage should be considered if anticipated blood volume loss is likely to result in transfusion in maternity patients and who are at increased risk of bleeding or in cases where allogenic blood transfusion is difficult or impossible.^8,9^ Cell salvage requires a local procedural guideline that should include patient selection, use of equipment, and reinfusion. All staff operating cell salvage devices should receive appropriate training, to ensure that they are familiar with and proficient in the technique. Additionally, in Rh D-negative maternity patients receiving salvaged blood where the cord blood group is Rh D-positive, a dose of Rh D immunoglobulin is required with additional doses based on the result of assessment of fetomaternal haemorrhage test.^8^

### Interventional Radiology

Preventive or therapeutic interventional radiology (IR) might have a role as a non-surgical approach. Potential role of preventive IR in improving the outcome of women (n = 958) undergoing surgery for a placenta accreta spectrum (PAS) disorder was systematically reviewed in 15 studies. Mean EBL was significantly lower in women who underwent preventive IR before surgery than those who did not (*P*  = .02). The EBL and the number of transfused FFP in pregnancies complicated with PAS who had an endovascular IR prior to hysterectomy were significantly lower than that of control.^24^ Current available data on preventive IR procedures are associated with lower EBL and need for transfusion in pregnant women with PAS scheduled for surgery.^24^

### Peripartum Hysterectomy

Although implementation of conservative approaches such as 1-step surgery or triple P procedure and/or its modifications or the intentional retention of the placenta have been recently recommended. To avoid severe maternal morbidity and mortality, peripartum hysterectomy (elective or emergency), which has been shown as a gold standard surgical management for PAS, still remains ultimate life-saving treatment.^25^ For successful surgical and analgesic/anaesthetic management of primary and secondary PPH with intensive care unit follow-up, pharmacological and/or interventional therapies are recommended.^26,27^

## Conclusion

Consequently, algorithms that include trigger and target values are to be used under the guidance of POC and/or standard laboratory tests. Primarily, the protocol should include strict attention to the control of bleeding, physiological and metabolic parameters, and active-early attempts to provide temperature maintenance. Secondly, focusing on fibrinogen which has a central role in coagulation to avoid unnecessary blood/blood product use that can increase mortality and/or morbidity should be kept in mind.

## Figures and Tables

**Table 1.** General Approach According to Estimated Blood Loss (EBL)

EBL 1000-1500 mL	EBL > 1500 mL
Call for help but do not leave the patient Position flat Apply O_2_ 15 L min^−1^ Assess patient (ABCDE) Monitor continuously Give 1 g of IV TXA (second dose in ongoing bleeding) Establish 2 IV accesses (14-16 G) Administer crystalloids Collect 20 mL of blood for laboratory/POC tests Notify blood bank and interventional radiology suite Consider surgery-anaesthesia/transfer to operating room Perform bimanual uterine massage-administer uterotonics	Monitor laboratory and/or POC tests including complete blood count (CBC), biochemistry, fibrinogen, PT, APTT, lactate Monitor non-invasive cardiac output (if available) Consider invasive arterial/central monitoring Maintain haemodynamics—sustain normovolaemia Administer blood/blood products (if haemodynamics is unstable) Repeat 1 g of IV TXA Place uterine balloon tamponade Continue pharmacotherapy of uterotonics Optimise oxygenation and acid–base status Provide calcium homeostasis Prevent hypothermia Perform hysterectomy Consider intraoperative cell salvage (if available)

POC, point-of-care; TXA, tranexamic acid; IV, intravenous; ABCDE, airway, breathing, circulation, disability and exposure.

**Table 2.** Blood/Blood Product and Factor Concentrate Replacement Therapy for the Management of Postpartum Haemorrhage with Administration Timing and Dose Recommendations^2,14,15^

Blood/Blood Product and Factor Concentrates	Dose	When to Administer
RBC	Initial number of units depends on haemodynamics and EBL	Hb <7 g dL^−1^ during non-massive bleeding
		Hb: 7-9 g dL^−1^ during active bleeding
FFP	15-20 mL kg^−1^	APTT and PT/international normalized ratio (INR) 1.5 times of normal or EXTEM CT > 75 seconds
Platelet concentrate	5-10 mL kg^−1^	Platelet count <50 000 µL^−1^
Cryoprecipitate	4-6 mL kg^−1^	Fibrinogen concentration ≤2 g dL^−1^ or FIBTEM A5 ≤ 12 mm
Fibrinogen concentrate	25-50 mg kg^−1^ (1 g in 50 mL injection water)	Fibrinogen concentration ≤ 2 g dL^−1^ or FIBTEM A5 ≤ 12 mm
rFVIIa (off label)	60 or 90 µg kg^−1^	Life-threatening haemorrhage which cannot be stopped by conventional, surgical, or interventional radiological means and/or comprehensive coagulation therapy fails

RBC, red blood cell; FFP, fresh-frozen plasma; rFVIIa, recombinant activated factor VII; EBL, estimated blood loss; Hb, haemoglobin; EXTEM CT, ­clotting time; EXTEM, Activation of clot formation by thromboplastin (tissue factor); FIBTEM, Activation as in EXTEM with the addition of cytochalasin D, a platelet blocking substance. In the FIBTEM assay, fibrinogen levels and fibrin polymerisation can be assessed in a functional way.
